# Supplementary material for: Injection of Ultra-Purified Stem Cells with Sodium Alginate Reduces Discogenic Pain in a Rat Model
Source: Cells. 2023 Feb 3;12(3):505. doi: 10.3390/cells12030505 (PMC9914726; doi:10.3390/cells12030505)
Supplement: Supplementary file 1 [file cells-12-00505-s001.zip › cells-2174904-supplementary.pdf]

cells

MDPI

Supplementary Materials

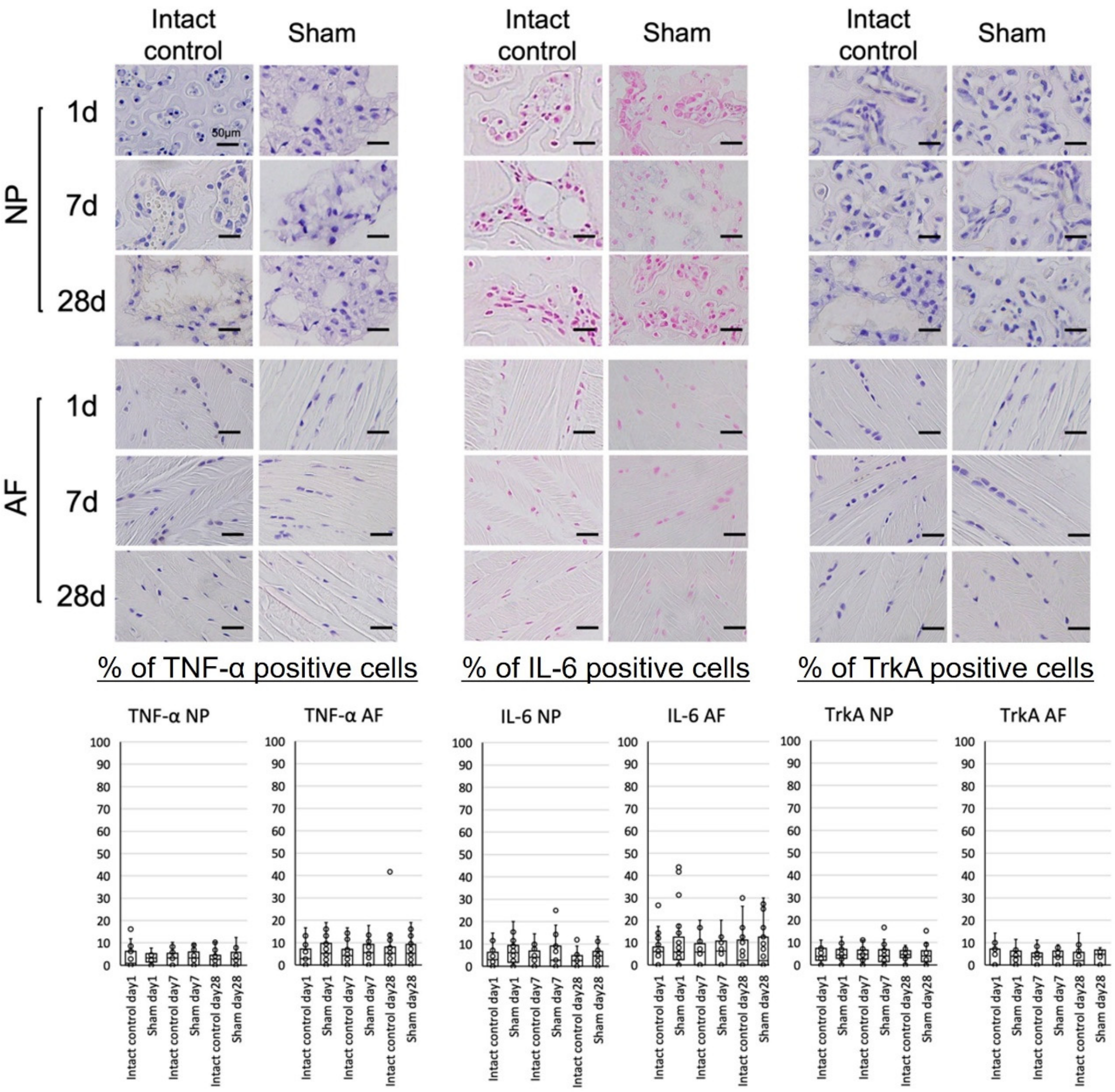

Figure S1. Immunohistochemistry (IHC) analysis in the groups of intact control and sham. Representative immunohistochemistry (IHC) staining for tumor necrosis factor alpha (TNF-α), interleukin-6 (IL-6) and tyrosine kinase A (TrkA) of nucleus pulposus (NP) and annulus fibrosus (AF) tissues on postoperative days 1, 7, and 28. There were no significant differences between the intact control and the sham groups in all respects. The boxes represent the median and the interquartile range, with the vertical lines showing the range (at each time point, n=3 rats; n=6 IVDs in each group).

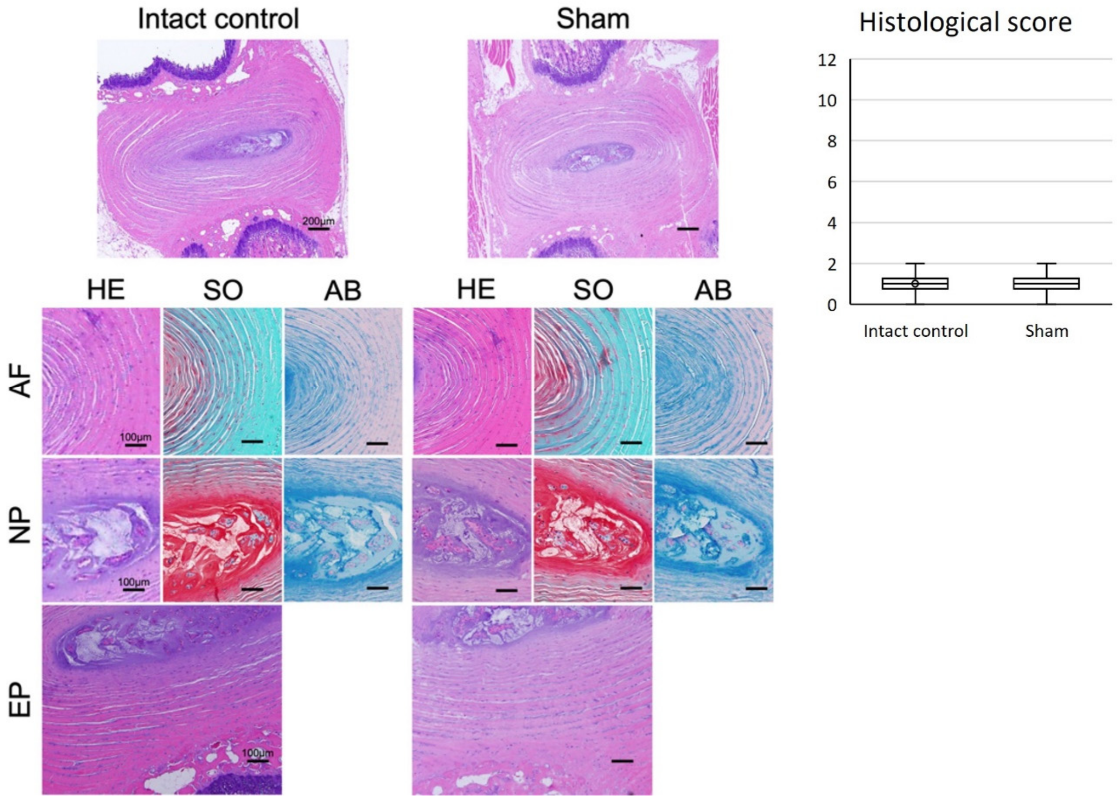

Figure S2. Histological analysis in the groups of intact control and sham. Representative image of Haematoxylin and eosin (HE), Safranin O (SO), and Alcian blue (AB) staining in annulus fibrosus (AF), nucleus pulposus (NP), and endplate (EP) tissues. The histological degeneration score in the intact control group was equivalent to those in the sham group. The boxes represent the median and the interquartile range, with the vertical lines showing the range (at each time point, n = 3 rats; n = 6 IVDs in each group).

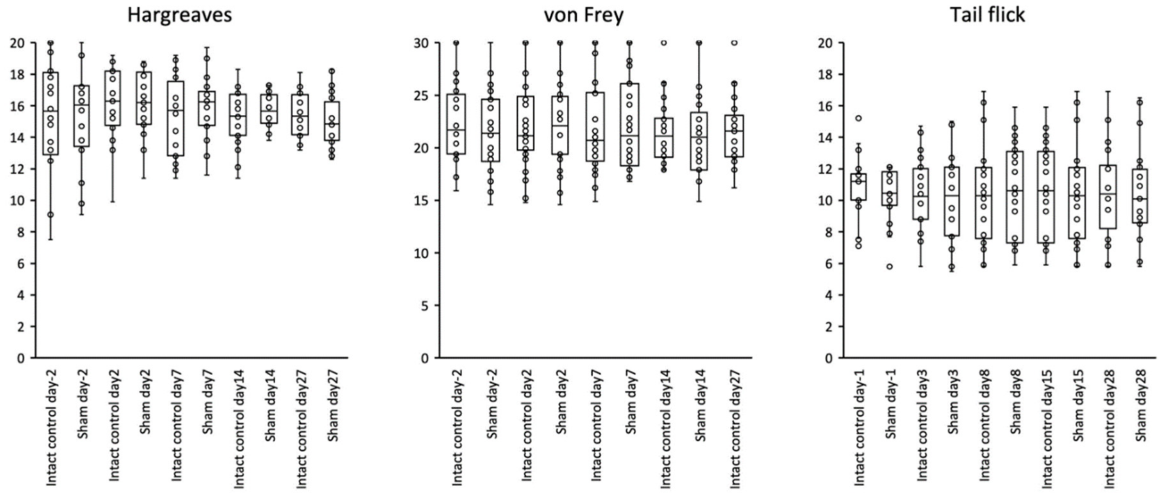

Figure S3. Behavioral nociception assays in the groups of intact control and sham. The Hargreaves and von Frey tests were performed 2 days before surgery (day −2) and on days 2, 7, 14, and 27 after surgery. The tail-flick test was performed 1 day before surgery (day −1) and on days 3, 8, 15, 28 after surgery. There were no significant differences in withdrawal latency between the intact control and the sham groups. The boxes represent the median and the interquartile range, with the vertical lines showing the range (at each time point, n = 6 rats in each group).

Table S1. Breakdown of histological degeneration score.

Category	Score			
	Sham	Punch	UPAL	REC+UPAL
Endplate	0.17 ± 0.17	1.33 ± 0.21	0.17 ± 0.17	0.17 ± 0.17
AF	0	1.67 ± 0.21	0.17 ± 0.17	0
Boundary between NP and AF	0	2	0.17 ± 0.17	0
Cellularity of NP	0.17 ± 0.17	1.5 ± 0.22	0.17 ± 0.17	0.17 ± 0.17
Matrix NP(H&E)	0	1.83 ± 0.17	0.17 ± 0.17	0.17 ± 0.17
Matrix NP(SO)	0.67 ± 0.21	1.33 ± 0.21	0.67 ± 0.21	0.67 ± 0.21
Total score	1 ± 0.26	9.66 ± 0.42	1.5 ± 0.22	1.17 ± 0.31

N=6 discs in each group, mean ± standard deviation.
MP; nucleus pulposus. AF; annuls fibrosus. H&E; hematoxylin and eosin. SO; safranin O
